# Mass cytometry revealed the circulating immune cell landscape across different Suzuki stages of Moyamoya disease

**DOI:** 10.1007/s12026-024-09464-x

**Published:** 2024-02-20

**Authors:** Chenglong Liu, Peicong Ge, Bojian Zhang, Liujia Chan, Yuheng Pang, Chuming Tao, Junsheng Li, Qiheng He, Wei Liu, Siqi Mou, Zhiyao Zheng, Zhikang Zhao, Wei Sun, Qian Zhang, Rong Wang, Yan Zhang, Wenjing Wang, Dong Zhang, Jizong Zhao

**Affiliations:** 1 Department of Neurosurgery, Beijing Tiantan Hospital, Capital Medical University, Beijing, 100070 China; 2 China National Clinical Research Center for Neurological Diseases, Beijing, 100070 China; 3 Beijing Institute of Hepatology, Beijing YouAn Hospital, Capital Medical University, Beijing, 100069 China; 4 Department of Neurosurgery, Beijing Hospital, National Center of Gerontology, Beijing, 100730 China; 5 Institute of Geriatric Medicine, Chinese Academy of Medical Sciences, Beijing, 100730 China

**Keywords:** Moyamoya disease, Suzuki stage, Mass cytometry, Circulating immune cell, NF-κB

## Abstract

Moyamoya disease (MMD) is a cerebrovascular disorder marked by progressive arterial narrowing, categorized into six stages known as Suzuki stages based on angiographic features. Growing evidence indicates a pivotal role of systemic immune and inflammatory responses in the initiation and advancement of MMD. This study employs high-dimensional mass cytometry to reveal the immunophenotypic characteristics of peripheral blood immune cells (PBMCs) at various Suzuki stages, offering insights into the progression of MMD. PBMC samples from eight patients with early-stage MMD (Suzuki stages II and III) and eight patients with later-stage MMD (Suzuki stages IV, V, and VI) were analyzed using high-dimensional mass cytometry to evaluate the frequency and phenotype of immune cell subtypes. We identified 15 cell clusters and found that the immunological features of early-stage MMD and later-stage MMD are composed of cluster variations. In this study, we confirmed that, compared to later-stage MMD, the early-stage MMD group exhibits an increase in non-classical monocytes. As the Suzuki stage level increases, the proportions of plasmacytoid DCs and monocyte-derived DCs decrease. Furthermore, T cells, monocytes, DCs, and PMN-MDSCs in the early-stage MMD group show activation of the canonical NF-κB signaling pathway. We summarized and compared the similarities and differences between early-stage MMD patients and later-stage MMD patients. There is a potential role of circulating immune dysfunction and inflammatory responses in the onset and development of MMD.

**Supplementary Information:**

The online version contains supplementary material available at 10.1007/s12026-024-09464-x.

## Introduction

Moyamoya disease (MMD) is a cerebrovascular disorder characterized by chronic and progressive stenosis of the distal internal carotid arteries, proximal anterior cerebral arteries, and proximal middle cerebral arteries in both hemispheres, accompanied by the formation of abnormal vascular networks at the base of the skull [1, 2]. Cerebral angiography is considered the gold standard for diagnosing this condition [3]. In 1969, Japanese researchers Suzuki and Takaku classified the angiographic features of MMD into six stages based on the disease’s progression. This classification, known as the Suzuki stage, has become the widely accepted assessment standard for MMD, providing a relatively accurate reflection of the blood supply in the anterior circulation of MMD patients [4]. While previous studies have explored the relationship between hemodynamic features and surgical outcomes in different Suzuki stages [5, 6], research on the immune status of patients in various Suzuki stages and the potential mechanisms remains limited.

MMD may result from vascular immune injury and an inflammatory response [7]. Its primary pathological changes involve the proliferation of smooth muscle cells and extracellular matrix in narrowed arteries. As the disease progresses, the tunica media gradually becomes thinner in comparison to the significantly thickened intima [8]. Masuda and colleagues also observed that during the smooth muscle proliferation process in the narrowed arteries of MMD patients, there was infiltration of T lymphocytes and macrophages on the intimal surface, leading to intimal hyperplasia. This observation underscored the inflammatory mechanisms at play in MMD [9].

Previous research, involving bioinformatics analysis of tissue samples from the M4 segment of the middle cerebral artery, which is proximal to the onset site of MMD, has indicated significant differences in immune cell infiltration between MMD patients and control groups. This finding suggests a notable immune response specific to MMD, highlighting the potential role of immune mechanisms in the pathology of the disease [10]. Additionally, a close association was observed between dysregulated genes in MMD and genes related to autoimmune diseases [11]. Concerning peripheral immunity in MMD, previous single-cell sequencing has suggested that the interaction of peripheral immune cells may play a role in the pathogenesis of MMD [12]. Leukocytes are vital components of the immune system and play a central role in peripheral blood [13]. Currently, there is limited research on peripheral blood immune cells in MMD patients. Therefore, investigating the peripheral immune landscape in MMD patients at different stages of the disease can help enhance our understanding of its pathogenic mechanisms and provide comprehensive insight into the progression of this chronic condition.

In this study, we employed high-dimensional mass cytometry (CyTOF) to identify differences in peripheral blood immune cells among MMD patients. Our objective was to unveil the immunophenotypic features of peripheral blood immune cells in individuals with MMD at different Suzuki stages. The research provides a comprehensive characterization of these immune cell cluster phenotypes, offering potential insights into the identification of peripheral blood immune cells and a deeper understanding of the mechanisms underlying MMD.

## Materials and methods

### Study design and participants

This study included 16 patients with MMD from the Department of Neurosurgery at Beijing Tiantan Hospital, Capital Medical University. According to the 2021 Guidelines published in Japan, the diagnosis of MMD is typically confirmed through digital subtraction angiography [14]: (1) stenosis or occlusion at the terminal portion of the internal carotid artery and the proximal portion of the anterior and middle cerebral arteries and (2) either one side (unilateral) or both sides (bilateral) are affected. Patients with concurrent autoimmune diseases were excluded from the study. The study received approval from the institutional review committee, and informed consent was obtained from the patients or their representatives. Moreover, we adhered to all relevant national and international data protection regulations, ensuring that the privacy of the participants and the integrity of the data were comprehensively protected.

### Data collection

MMD patients’ clinical characteristics obtained at admission include baseline data (age and gender), clinical features (heart rate, blood pressure, and body mass index), medical history (hypertension, diabetes, dyslipidemia, smoking status, and alcohol consumption), and initial clinical presentations (ischemic type and hemorrhagic type). After a 15-min rest, the subjects had their right arm’s systolic blood pressure (SBP) and diastolic blood pressure (DBP) measured using a standard mercury sphygmomanometer. Simultaneously, heart rate measurements were recorded using an electrocardiogram (ECG). Body mass index (BMI) was calculated as weight (in kg) divided by height (in m^2^).

In this study, we employed Suzuki’s angiographic stage to demonstrate the vascular imaging characteristics of MMD patients.^4^ DSA files from each hemisphere were independently reviewed and staged by two neurosurgeons who were blinded to the clinical data. Any inconsistencies were resolved through consultation with senior supervisors and thorough discussions during roundtable conferences with the Academic Committee. The Suzuki stage for MMD patients was defined based on the more severely affected side. Patients with Suzuki stages equal to or less than 3 were classified into the early-stage MMD group, while those with stages greater than 3 were categorized into the later-stage MMD group.

### Mass cytometry

Blood samples were obtained from the patients’ peripheral veins after an 8-h fasting period and collected into EDTA tubes, with processing within 2 h. Peripheral blood mononuclear cells (PBMCs) were separated from the blood samples using the Ficoll-Paque density gradient centrifugation method and stored at − 80 °C for further analysis. Supplementary Table 1 online provides a detailed description of the monoclonal antibodies used in our study. We washed the cells with PBS (containing 2% fetal bovine serum) and assessed cell viability using cisplatin staining following the manufacturer’s instructions. Subsequently, PBMCs were stained with metal-conjugated surface antibodies. After surface marker staining, the cells were washed. Following this, intracellular staining was performed on the PBMCs. After intracellular staining, the cells were washed twice and then stained with a DNA intercalator solution. Data acquisition was carried out using the Helios mass cytometer (Fluidigm, USA). Additionally, we detected the *RNF213* p.R4810K variant using the primers RNF213-4810F 5′-GCCCTCCATTTCTAGCACAC-3′ and RNF213-4810R 5′-AGCTGTGGCGAAAGCTTCTA-3′.

### Data analysis

A manual gating strategy was applied to each sample, and data for individual viable cells were extracted from the cell repository after preprocessing. Subsequently, a reverse hyperbolic sine (arcsinh) normalization method was applied to accurately identify clustering patterns. Following that, the high-dimensional data were analyzed using PhenoGraph and the R-package “cytofkit (v1.10.0)” with default parameters [15]. High-dimensional data visualization using *t*-Stochastic Neighbor Embedding (*t*-SNE) was performed to elucidate the overall patterns of clustering frequency, clustering heterogeneity, and marker expression that distinguish the two groups. The parameters were configured as follows: perplexity was set to 30,900 iterations were performed, and theta was set to 0.5. Clusters with similar phenotypic features were manually merged. Data on marker expression levels and cell counts were extracted from each cluster. Statistical analysis was conducted using the R packages “pheatmap (v1.0.12)” and “ggplot2 (v3.4.0)”.

We performed statistical analysis using SPSS version 26.0. A complete case analysis was conducted for all enrolled participants. Categorical variables were presented as frequencies, while continuous variables were presented as mean ± standard deviation (SD). Group comparisons were made using the *χ*^2^ test for categorical data and the Wilcoxon test for continuous data. The Wilcoxon rank-sum test was utilized to compare marker expression levels and cell counts. Kendall’s correlation test was employed to assess the correlation between the proportions of immune cells and the Suzuki stages. Differences were considered statistically significant when the two-tailed *p*-value was less than 0.05.

## Results

### Demographic and clinical characteristics of patients with MMD

A comparison of clinical and demographic characteristics between early-stage and later-stage MMD patients is presented in Table 1. There were eight patients in the early-stage MMD group, with an average age of 36.25 ± 9.72 years, and eight patients in the later-stage MMD group, with an average age of 42.00 ± 6.89 years. In the early-stage group, the gender ratio was five females to three males, while in the later-stage group, it was six females to two males. Age, gender, clinical features (heart rate, SBP, DBP, and BMI), vascular risk factors (hypertension, hypercholesterolemia, and diabetes mellitus), smoking and drinking history, *RNF213* p.R4810K mutation status, and clinical type showed no statistically significant differences between the two groups (*P* > 0.05).

**Table 1:** Demographic and clinical characteristics in patients with MMD

Characteristics	Early stage (*n* = 8)	Later stage (*n* = 8)	*P* value
Age, y, mean ± SD	36.25 ± 9.72	42.00 ± 6.89	0.207
Female/male ratio	5/3	6/2	1.000
Clinical features, mean ± SD			
Heart rate, bpm	81.38 ± 5.92	79.75 ± 4.74	0.491
SBP, mmHg	129.88 ± 4.39	128.63 ± 9.24	0.752
DBP, mmHg	79.63 ± 7.63	81.63 ± 9.12	0.713
BMI, kg/m^2^	27.30 ± 3.82	24.87 ± 1.68	0.345
*RNF213* p.R4810K, *n* (%)			1.000
Wild-type	6 (75.0)	7 (87.5)	
Mutant	2 (25.0)	1 (12.5)	
Vascular risk factors			
Hypertension	1 (12.5)	4 (50.0)	0.282
Hypercholesterolemia	3 (37.5)	0 (0.0)	0.200
Diabetes mellitus	0 (0.0)	0 (0.0)	1.000
Current cigarette smoking	2 (25.0)	1 (12.5)	1.000
Current alcohol	2 (25.0)	0 (0.0)	0.467
Clinical type, *n* (%)			0.315
Ischemic type	6 (75.0)	3 (37.5)	
Hemorrhagic type	2 (25.0)	5 (62.5)	

Early stage: in Suzuki stages II and III; later stage: in Suzuki stages IV, V, and VI

*MMD* moyamoya disease, *SD* standard deviation, *SBP* systolic blood pressure, *DBP* diastolic blood pressure, *BMI* body mass index

### High-dimensional cytometry profiling of PBMCs in early- and later-stage MMD: cluster analysis and molecular expression patterns

**Fig. 1:**
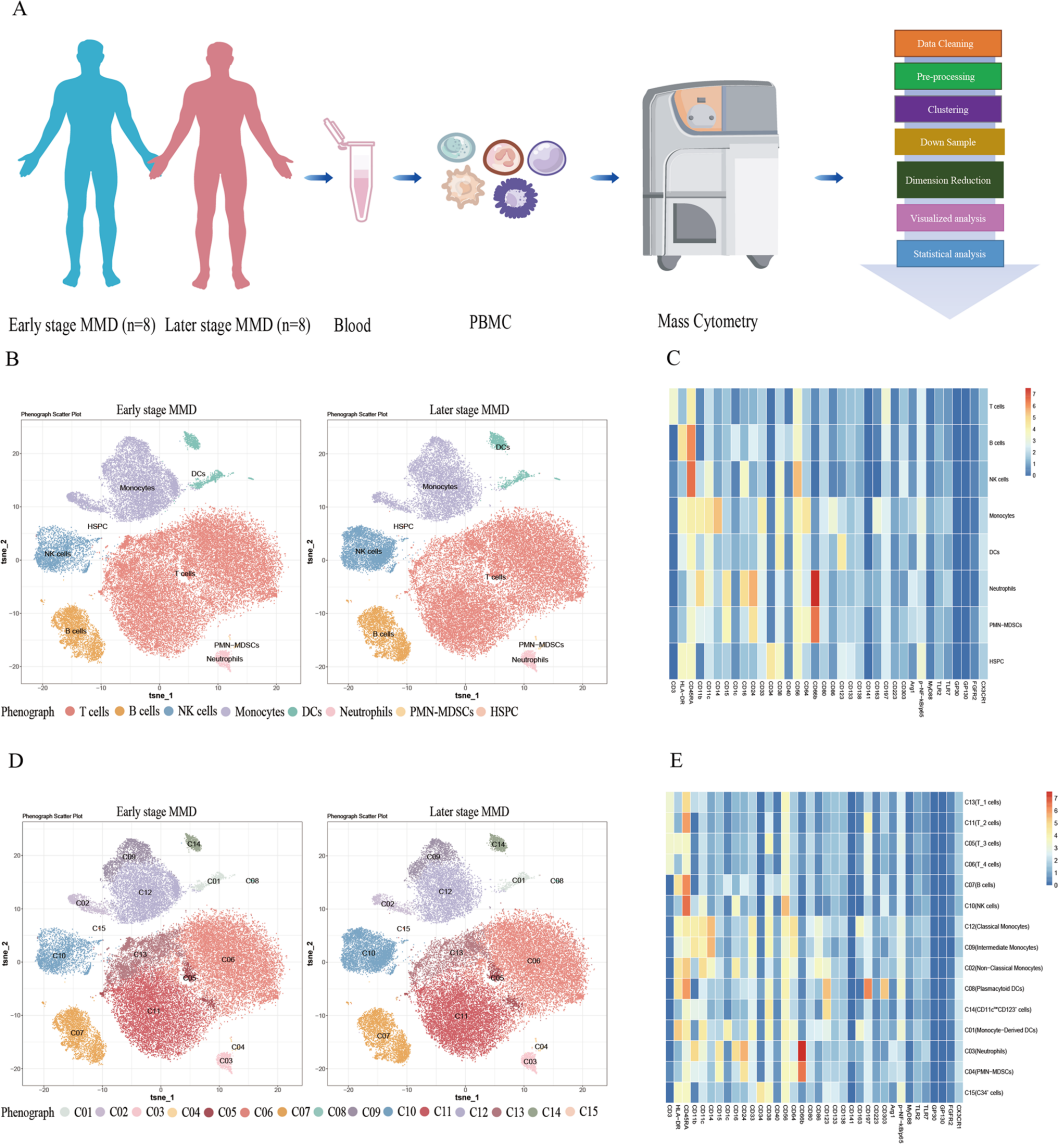
Overview of imaging mass cytometry study of early-stage and later-stage MMD. **A** Flowchart detailing the collection and analysis of PBMCs. **B** Representative *t*-SNE plot of eight cell clusters from early-stage and later-stage MMD patients, including T cells, B cells, NK cells, monocytes, dendritic cells (DCs), neutrophils, polymorphonuclear myeloid-derived suppressor cells (PMN-MDSCs), and hematopoietic stem and progenitor cells (HSPC). **C** Heatmap illustrating the expression of each protein in eight clusters of cells. **D** Representative *t*-SNE plot of fifteen cell clusters from early-stage and later-stage MMD patients, categorized as follows: T cells (four clusters: C05, C06, C11, C13), B cells (one cluster: C07), NK cells (one cluster: C10), monocytes (three clusters: C02, C09, C12), DCs (three clusters: C01, C08, C14), neutrophils (one cluster: C03), PMN-MDSCs (one cluster: C04), and HSPC (one cluster: C15). **E** Heatmap presenting the expression of each protein in the 15 subgroups of CD45^+^ cells. Early-stage refers to Suzuki stage II and III, while later-stage refers to Suzuki stages IV, V, and VI

Our high-dimensional cytometry profiling was conducted on PBMC samples obtained from eight early-stage MMD patients and eight later-stage MMD patients (Fig. 1A). Based on the expression patterns of the markers, we discriminated CD45^+^ PBMCs into 15 phenograph clusters using CyTOF. To delve further into the molecular expression of the circulating immune populations, we combined 15 phenograph clusters into eight merge clusters, encompassing T cells, B cells, NK cells, monocytes, dendritic cells (DCs), neutrophils, polymorphonuclear myeloid-derived suppressor cells (PMN-MDSCs), and hematopoietic stem and progenitor cells (HSPC). The comparison of phenotypic clusters for these 8 or 15 cell clusters with manually gated cell phenotypes is presented in Table 2. Figure 1B and D depict the landscapes of 15 subgroups and 8 subgroups of circulating immune cells with reduced dimensionality by *t*-SNE, respectively. Figure 1C and E display heatmaps illustrating the molecular expression levels for 15 circulating immune cell subgroups and 8 circulating immune cell subgroups, respectively.

**Table 2:** Comparison of PhenoGraph clusters with manually gated cell phenotypes

Cell lineages	Cell type	PhenoGraph cluster	Clusters
T cells	CD45RA^high^HLA-DR^−^CD38^−^CD11b^+^	C13	T_1 cells
	CD45RA^high^HLA-DR^−^CD38^+^CD11b^−^	C11	T_2 cells
	CD45RA^low^HLA-DR^+^CD38^+^CD11b^−^	C05	T_3 cells
	CD45RA^low^HLA-DR^−^CD38^−^CD11b^−^	C06	T_4 cells
B cells	CD24^+^CD40^+^CD1c^+^	C07	B cells
NK cells	CD56^+^CD16^+^	C10	NK cells
Monocytes	CD14^++^CD16^−^	C12	Classical monocytes
	CD14^++^CD16^+^	C09	Intermediate monocytes
	CD14^+^CD16^++^	C02	Non-classical monocytes
DCs	CD11c^low^CD123^+^CD303^+^	C08	Plasmacytoid DCs
	CD11c^low^CD123^+^CD303^−^	C14	CD11c^low^CD123^+^ cells
	CD11c^+^CD1c^+^HLA-DR^+^	C01	Monocyte-derived DCs
Neutrophils	CD14^−^CD15^+^CD16^+^CD66b^+^	C03	Neutrophils
PMN-MDSCs	CD14^−^CD15^+^CD16^−^CD66b^+^	C04	PMN-MDSCs
HSPC	C34^+^	C15	C34^+^ cells

*DCs* dendritic cells, *HSPC* hematopoietic stem and progenitor cells, *NK* natural killer, *PMN-MDSCs* polymorphonuclear myeloid-derived suppressor cells

**Fig. 2:**
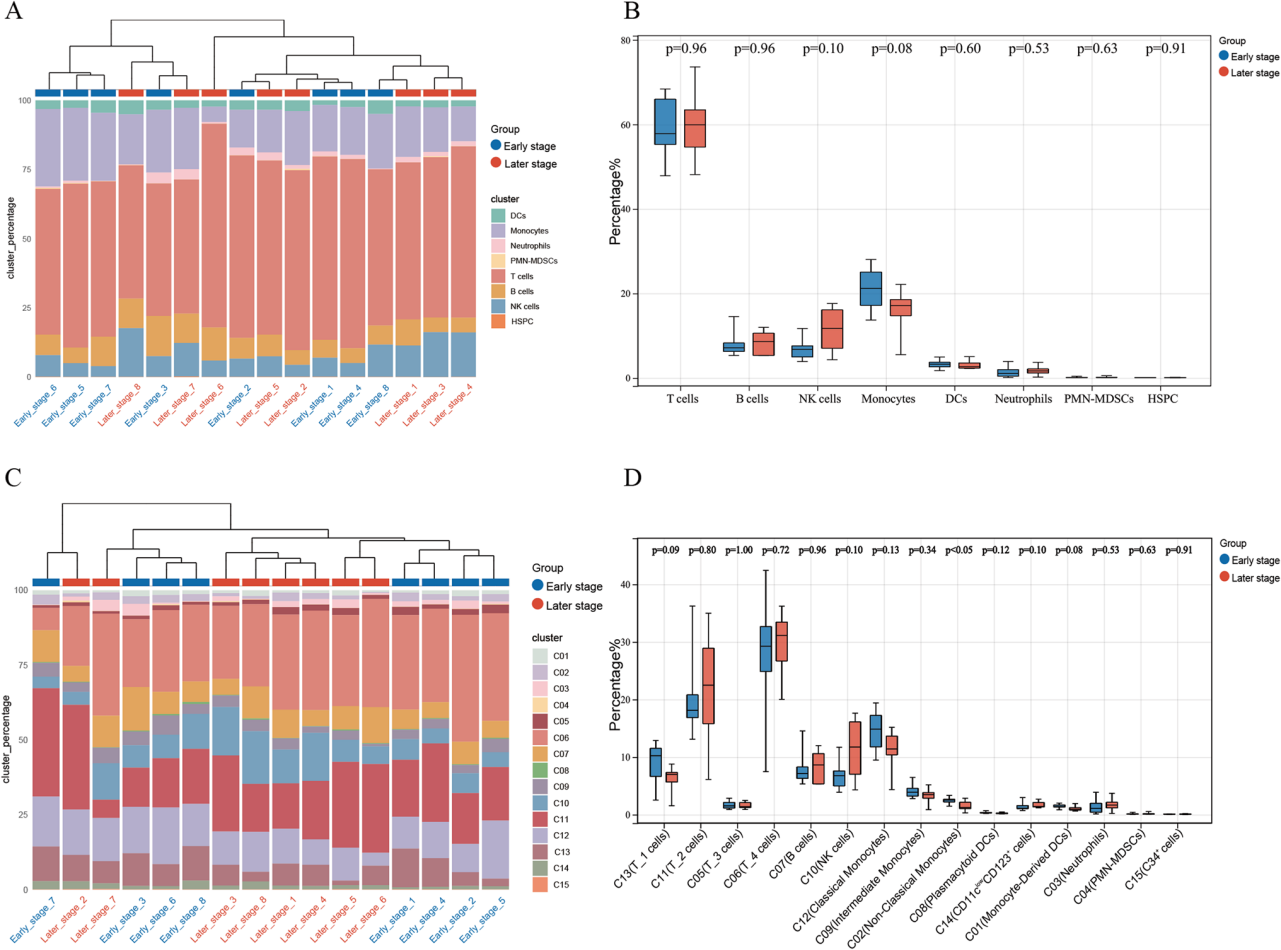
Proportions of CD45^+^ cell subgroups in early-stage and later-stage MMD. **A** Cluster diagrams illustrating the proportions of eight distinct cell types in both early-stage and later-stage MMD patients. **B** Percentage distribution of the eight subgroups of CD45^+^ cells in early-stage (*n* = 8) and later-stage (*n* = 8) MMD patients. **C** Cluster diagrams depicting the proportions of 15 different cell types in both early-stage and later-stage MMD patients. **D** Percentage distribution of the 15 subgroups of CD45^+^ cells in early-stage (*n* = 8) and later-stage (*n* = 8) MMD patients. *P* values were calculated using the two-sided Wilcoxon test, and *P* value of less than 0.05 was considered statistically significant

When we divided circulating immune cells into eight subgroups based on differences in molecular expression levels, Fig. 2A shows the proportions of cell subpopulations in each sample. No significant differences in the percentage of the eight cell lineages were observed between the early-stage and later-stage groups (*P* all > 0.05, Fig. 2B). Subsequently, we divided circulating immune cells into 15 subgroups based on differences in molecular expression levels; Fig. 2C shows the proportions of cell subpopulations in each sample. Among the three clusters (C02, C09, and C12) of monocytes, the percentage of C02 (non-classical monocytes) was lower in the later-stage group compared to the early-stage group (*P* < 0.05). No statistical differences were observed in the other cell clusters (Fig. 2D).

### Linear correlation between Suzuki’s angiographic stage and cell cluster proportions

**Fig. 3:**
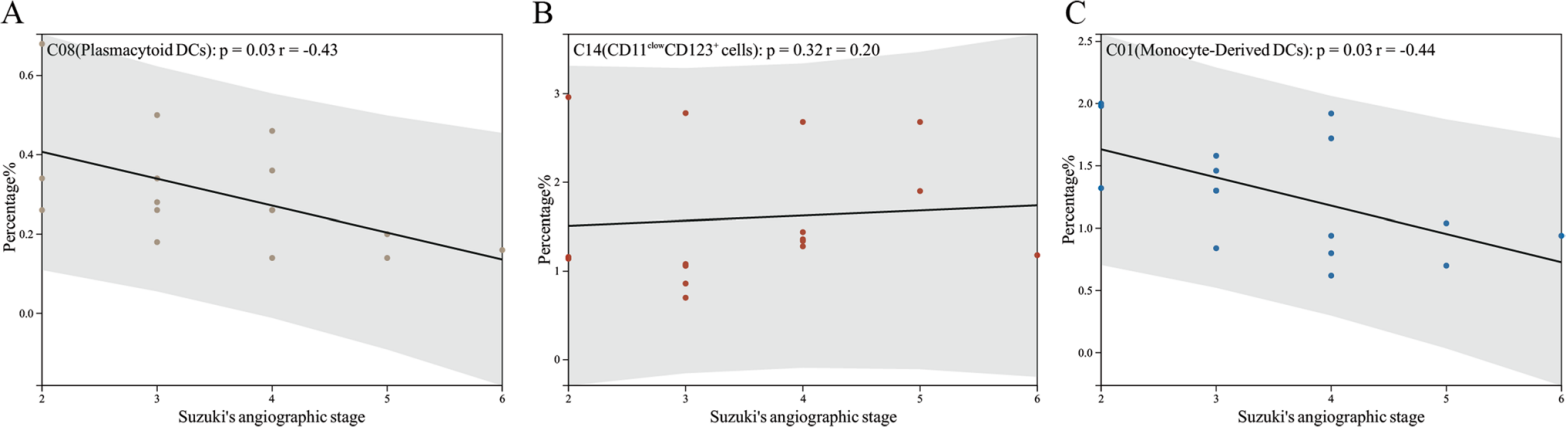
Kendall correlation analysis between Suzuki’s angiographic stages and proportions of DC clusters.** A** C08 (plasmacytoid DCs), **B** C14(CD11c^low^CD123^+^ cells), and **C** C01 (monocyte-derived DCs). The analysis reveals insights into the relationships between angiographic stages and specific dendritic cell clusters. Statistical significance was set at *P* < 0.05

With the increase in Suzuki’s angiographic stage, there is no linear change in the cell proportions of T cells, B cells, NK cells, monocytes, and DCs (*P* all > 0.05, Supplementary Fig. 1). However, in three DCs clusters (C01, C08, and C14), the proportions of C01 (monocyte-derived DCs) and C08 (plasmacytoid DCs) decrease linearly with the increase in Suzuki’s angiographic stage, with significant correlations for both C01 (*P* = 0.03, *r* =  − 0.44) and C08 (*P* = 0.03, *r* =  − 0.43) in Fig. 3. The cell proportions of the other clusters do not show a linear change with Suzuki’s angiographic stage (*P* all > 0.05; Supplementary Fig. 2).

### Differential expression of FGFR2, TLR2, TLR7, MyD88, p-NF-κb/p65, CX3CR1, and CD197 in early- and later-stage MMD groups across cell clusters

**Fig. 4:**
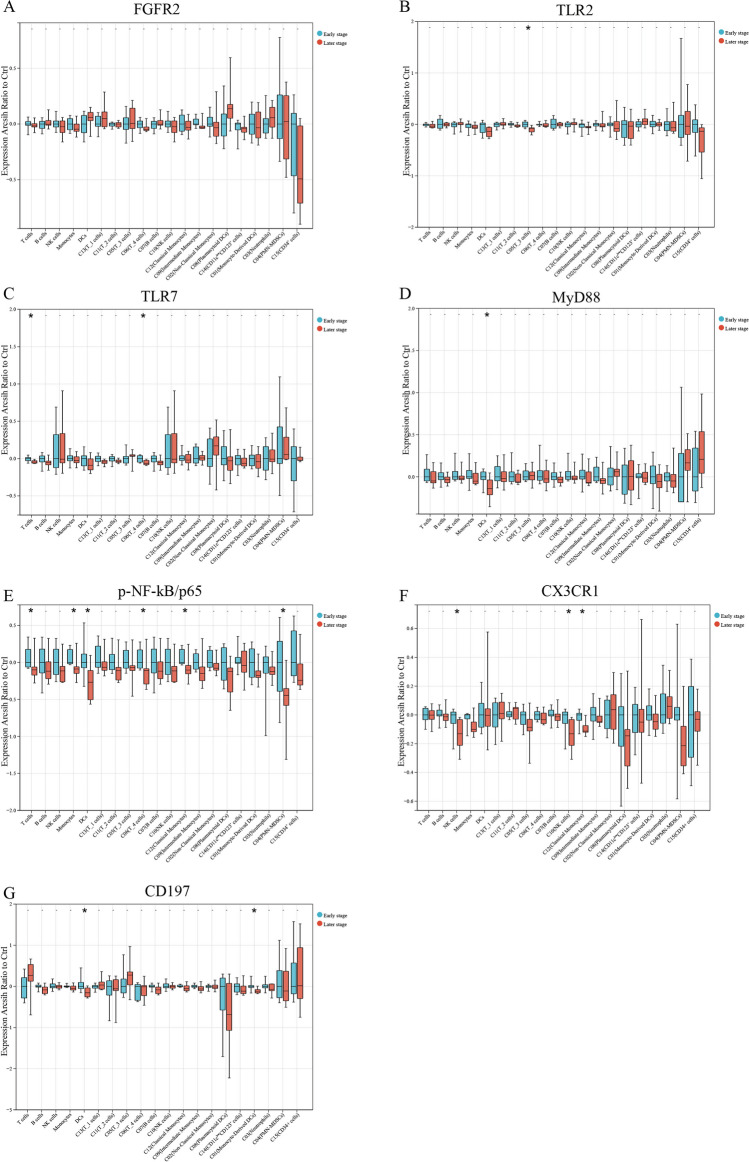
Molecular expression of key markers in circulating immune cells in early-stage and later-stage MMD. **A** Expression levels of FGFR2 in circulating immune cells.** B** Expression levels of TLR2 in circulating immune cells. **C** Expression levels of TLR7 in circulating immune cells.** D** Expression levels of MyD88 in circulating immune cells. **E** Expression levels of p-NF-kB/p65 in circulating immune cells. **F** Expression levels of CX3CR1 in circulating immune cells. **G** Expression levels of CD197 in circulating immune cells. Sample size: early-stage MMD (*n* = 8), later-stage MMD (*n* = 8). Statistical analysis was performed using the two-sided Wilcoxon test, and a *P* value of less than 0.05 was considered statistically significant (*P < 0.05)

The expression levels of fibroblast growth factor receptor 2 (FGFR2) did not differ statistically between the early-stage and the later-stage MMD group across the five cell clusters and 15 subclusters (Fig. 4A). In the early-stage MMD group, Toll-like receptor 2 (TLR2) showed higher expression in the C05 (T_3 cells) cluster compared to the later-stage MMD group, with no statistically significant differences in other clusters (Fig. 4B). Additionally, the early-stage MMD group exhibited elevated expression of Toll-like receptor 7 (TLR7) in T cells and the C06 (T_4 cells) cluster compared to the later-stage MMD group, with no significant differences observed in other clusters (Fig. 4C). In DCs, the early-stage MMD group showed higher expression of myeloid differentiation factor 88 (MyD88) compared to the later-stage MMD group, with no statistically significant differences observed in other clusters (Fig. 4D).

Furthermore, the early-stage MMD group demonstrated higher levels of p-NF-κb/p65 expression in T cells, monocytes, DCs, C04(PMN-MDSCs), C06(T_4 cells), and C12 (classical monocytes) compared to the later-stage MMD group (Fig. 4E). Additionally, the expression of CX3CR1 in NK cells and C12 (classical monocytes) cell clusters was higher in the early-stage MMD group than in the later-stage MMD group (Fig. 4F). Moreover, the early-stage MMD group showed higher expression of CD197 in DCs and C01 (monocyte-derived DC) cell clusters compared to the later-stage MMD group (Fig. 4G).

### Molecular expression in DCs

**Fig. 5:**
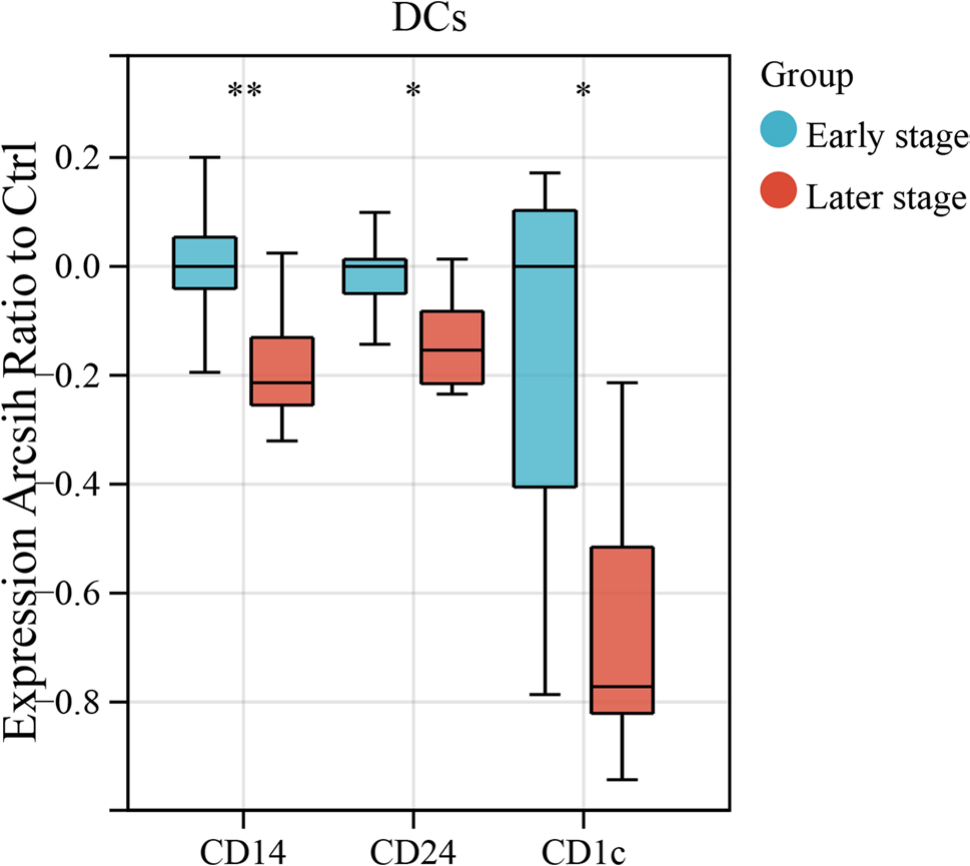
Molecular expression of DCs. This figure compares the molecular expression of DCs in early-stage MMD (*n* = 8) with that in later-stage MMD (*n* = 8). *P* values were calculated using the two-sided Wilcoxon test. Significance: *P < 0.05; **P < 0.01

In comparison to the later-stage group, the early-stage group showed elevated levels of CD14, CD24, and CD1c molecule expression (Fig. 5).

## Discussion

The onset and progression of MMD are associated with immune dysregulation and inflammatory responses in the body [16, 17]. Studies have indicated an increased risk of MMD with elevated systemic immune and inflammatory markers [18]. Previous investigations have utilized samples from MMD patients, such as cerebrospinal fluid or peripheral blood, to identify specific molecules, including autoantibodies and various RNA molecules [19–22]. CyTOF, as an advanced cell analysis technology, offers multiple advantages, including high dimensionality, low noise, and multi-parameter analysis, making it suitable for a wide range of biomedical research areas [23, 24]. In a previous study, our team pioneered the use of CyTOF to reveal distinctions in the peripheral immune landscape between healthy individuals and those with unruptured intracranial aneurysms. This work uncovered potential roles for dysfunctions in circulating immune functions in the development of unruptured intracranial aneurysms, indicating a significant link between immune system behavior and the pathophysiology of these aneurysms [25]. However, its application in the field of cerebrovascular diseases, particularly in MMD research, has been limited. In this study, we confirmed that, compared to later-stage MMD, the early-stage MMD group exhibits an increase in non-classical monocytes. As the Suzuki stage level increases, the proportions of plasmacytoid DCs and monocyte-derived DCs decrease. Furthermore, T cells, monocytes, DCs, and PMN-MDSCs in the early-stage MMD group show activation of the canonical NF-κB signaling pathway.

The Suzuki stage system is widely used to assess MMD, serving as a grading system to evaluate the anterior circulation condition of patients. MMD patients undergo a gradual narrowing and occlusion of major intracranial arteries from Suzuki stage I to stage III, accompanied by an increase in collateral vessels. From Suzuki stage IV to stage VI, the collateral vessels decrease, and the supply from the internal carotid arteries diminishes, leading to a gradual dependence on the external carotid or vertebral artery pathways for cerebral circulation [26]. During the disease progression, circulating monocytes may play a role in MMD by leaving the bloodstream, migrating through the endothelium, and differentiating into tissue macrophages. These tissue macrophages can initiate and facilitate further immune responses [9, 17]. Elevated levels of non-classical monocytes, as a distinctive subset, are associated with certain autoimmune diseases and viral infections [27, 28]. In our study, the increased levels of non-classical monocytes in the early-stage MMD group may be linked to a chronic inflammatory state. Additionally, the elevated expression of CX3CR1 in classical monocytes of early-stage MMD patients suggests an enhanced capacity for adhesion and chemotaxis.

DCs are known as highly functional antigen-presenting cells, bridging innate and adaptive immunity through antigen processing and presentation to T and B lymphocytes [29]. In our study, we observed a decrease in the proportion of plasmacytoid DCs and monocyte-derived DCs as Suzuki stages increased. This suggests that as the disease progresses, the extent of inflammation may decrease, leading to a relative reduction in these cell types. The decline in the proportion of DCs may result in a diminished capacity for antigen presentation in the body, and in the later stages of MMD, it could be associated with immune cell depletion. Additionally, in the early-stage MMD, DCs and monocyte-derived DCs exhibited higher expression levels of CD197 (CCR7), possibly indicating an enhanced migratory capacity of these cells to lymphoid tissues. At the same time, we also observed high expression of CD24 and CD1c in DCs of the early-stage MMD group. The increase in CD24 is typically associated with anti-inflammatory characteristics [30], while CD1c is closely related to antigen presentation and the activation of the immune response [31]. Elevated CD1c expression may enhance the antigen presentation capability of DC cells.

Fujimura et al. [32] and Young et al. [33] have reviewed the signaling cascade reactions and histology associated with moyamoya angiopathy, indicating an increase in transforming growth factor (TGF), hepatocyte growth factor (HGF), basic fibroblast growth factor (bFGF), and vascular endothelial growth factor (VEGF) in moyamoya angiopathy patients [34]. These growth factors may be related to angiogenesis and inflammation. In our study, FGFR2, implicated in pathways related to FGF, showed no differences in circulating immune cells. The impact of the FGF-related pathway on disease progression remains unclear and requires further experimental validation.

In our study, increased p-NF-κB/p65 was widely observed in peripheral immune cells, including T cells and T_4 cells, monocytes and classical monocytes, DCs, and PMN-MDSCs. The transcription factor NF-κB is a critical regulator of immune and inflammatory responses, operating through two distinct pathways: (1) the canonical pathway primarily activated by pathogens and inflammatory mediators and (2) the non-canonical pathway mostly activated by developmental cues. The most abundant form of NF-κB activated by pathological stimuli through the canonical pathway is the p65: p50 heterodimer [35]. The disproportionate increase in activated p65 and subsequent transcriptional activation of effector molecules are indispensable mechanisms in the pathogenesis of many chronic diseases, such as rheumatoid arthritis, inflammatory bowel disease, multiple sclerosis, and even neurodegenerative pathologies [36]. The induced activation of p65 in response to various stimuli is typically transient but sufficient to upregulate the transcription of various active target genes, including those involved in cell proliferation, inflammatory cytokines, chemokines, and apoptosis mediators [37, 38]. Research on the susceptibility gene *RNF213* in MMD indicates that genetic variations in the *RNF213* gene may induce NF-κB related inflammation, leading to vascular smooth muscle cell (VSMC) damage, a characteristic of the pathophysiology of MMD [39]. In our study, there was no statistically significant difference in the mutation rate of the *RNF213*p.4810 K site between the early-stage and later-stage MMD groups. However, for the early-stage MMD group, there was activation of the NF-κB pathway in circulating peripheral immune cells. This suggests that in MMD, the activation of the NF-κB pathway, unrelated to the mutation, may be involved in the progression of the disease.

Toll-like receptors (TLRs) are transmembrane proteins closely associated with both innate and adaptive immunity, playing a role in the pathogenesis of numerous inflammatory diseases [40]. The TLR family has two main signaling pathways: MyD88-dependent and MyD88-independent, with the MyD88-dependent pathway being common to all TLR signals except TLR3. The TLR family can activate the NF-κB signal pathway through MyD88, leading to increased release of pro-inflammatory cytokines and chemokines [41]. In our study, we observed that in the early-stage MMD group, the expression levels of TLR family members (TLR2 and TLR7) increased in T cells and their subsets (T_3 and T_4 cells). Additionally, there was an increase in p-NF-κB/p65 in T cells, indicating activation of the NF-κB pathway. Furthermore, in DCs, molecules related to the CD14/MyD88/NF-κB pathway showed an increased expression. It can be inferred that in the early stages of MMD, inflammation is closely associated with DCs. Bacterial lipopolysaccharides (high-affinity receptor for CD14) may act as triggers for inflammation, and the resulting activation of the NF-κB pathway is involved in the progression of the disease.

The flow cytometry antibodies we designed can separate neutrophils, PMN-MDSCs, and CD34^+^ cells from the peripheral CD45^+^ cell population. Previous studies have indicated an association between neutrophil-related genes and CD34^+^ cells with the onset of MMD [10, 42]. However, possibly due to our limited sample size, we did not observe significant differences between early and late stages of MMD, except in PMN-MDSCs (pathologically activated neutrophils), where activation of the NF-κB pathway was identified in the early-stage MMD group. Additionally, the increased expression of CX3CR1 in NK cells of the early-stage MMD group suggests enhanced adhesion and chemotaxis capabilities, indicating a more active role of NK cells in inflammation and immune responses in early-stage MMD patients.

While our study is the first to apply CyTOF in examining the differences in the peripheral immune landscape among subgroups of MMD patients, it does have limitations. Firstly, the small sample size necessitates further validation of our findings in a larger cohort to establish their significance. Secondly, the differences in peripheral immune cells and molecular signals found between early and later-stage MMD patients require additional basic research. This will facilitate their clinical application as potential therapeutic targets to delay the progression of MMD. Addressing these issues and expanding upon our research will be the focus of our future efforts.

## Conclusion

In this study, we revealed the landscape of circulating immune cells in subgroups of MMD (early and later stages), characterized by an increased number of non-classical monocytes in the early-stage MMD group and activation of the canonical NF-κB signaling pathway in T cells, monocytes, DCs, and PMN-MDSCs. Additionally, as the Suzuki stage level increased, there was a reduction in the proportions of plasmacytoid DCs and monocyte-derived DCs. Overall, this research elucidates the immune dynamics during the progression of MMD, providing insights into potential therapeutic targets and diagnostic markers.

### Supplementary Information

Below is the link to the electronic supplementary material.

Supplementary file1 (DOCX 464 KB)

## Data Availability

No datasets were generated or analyzed during the current study.
