# Supplementary material for: Mass cytometry revealed the circulating immune cell landscape across different Suzuki stages of Moyamoya disease
Source: Immunol Res. 2024 Feb 20;72(4):654–64. doi: 10.1007/s12026-024-09464-x (PMC11347468; doi:10.1007/s12026-024-09464-x)
Supplement: Supplementary file 1 — Supplementary file1 (DOCX 464 KB) [file 12026_2024_9464_MOESM1_ESM.docx]

| Antigen | Symbol and Mass | Antibody clone | Source |
| --- | --- | --- | --- |
| CD45 | 89Y | HI30 | Fluidigm |
| CD3 | 111Cd | Hu113 | R&D |
| p-NF-κB/p65 | 141Pr | 27.Ser 536 | Santa Cruz |
| CD40 | 142Ce | 5C3 | Fluidigm |
| MyD88 | 143Nd | EPR590(N) | Abcam |
| CD38 | 144Nd | HIT2 | Biolegend |
| CD16 | 145Nd | 3G8 | Fluidigm |
| TLR7 | 146Nd | 533707 | R&D |
| CD86 | 147Sm | IT2.2 | Biolegend |
| CD14 | 148Sm | M5E2 | Biolegend |
| CD34 | 149Sm | 581 | Biolegend |
| CD223 | 150Sm | 11C3C65 | Fluidigm |
| GP130 | 151Eu | EPR24052-38 | Abcam |
| CD24 | 152Gd | ML5 | Abcam |
| CD1c | 153Eu | EPR9638 | Abcam |
| CD197 | 154Gd | G043H7 | Biolegend |
| TLR2 | 155Gd | TL2.1 | Santa Cruz |
| HLA-DR | 156Gd | L243 | Biolegend |
| CD33 | 158Gd | WM53 | Biolegend |
| CD68 | 159Tb | KP1 | Fluidigm |
| CD64 | 160Dy | 10.1 | Biolegend |
| CD15 | 161Dy | SSEA-1 | Biolegend |
| CD11c | 162Dy | Bu15 | Biolegend |
| CD80 | 163Dy | 37711 | Fluidigm |
| Arg1 | 164Dy | D4E3M | Fluidigm |
| CD133 | 165Ho | 170411 | R&D |
| CD45RA | 166Er | HI100 | Fluidigm |
| CD303 | 167Er | BDCA-2 | Biolegend |
| CD56 | 168Er | A19063A | Biolegend |
| GP30 | 169Tm | EPR24052-38 | Abcam |
| FGFR2 | 170Yb | EPR24075-418 | Abcam |
| CD123 | 171Yb | 6H6 | Biolegend |
| CX3CR1 | 172Yb | K0124E1 | Fluidigm |
| CD141 | 173Yb | M80 | Biolegend |
| CD66b | 174Yb | 913542 | R&D |
| CD163 | 175Lu | EPR19518 | Abcam |
| CD138 | 176Lu | DL-101 | Biolegend |
| CD11b | 209Bi | ICRF44 | Fluidigm |

Supplementary Material

**Supplementary Table 1. Mass cytometry antibodies panel design.**

**
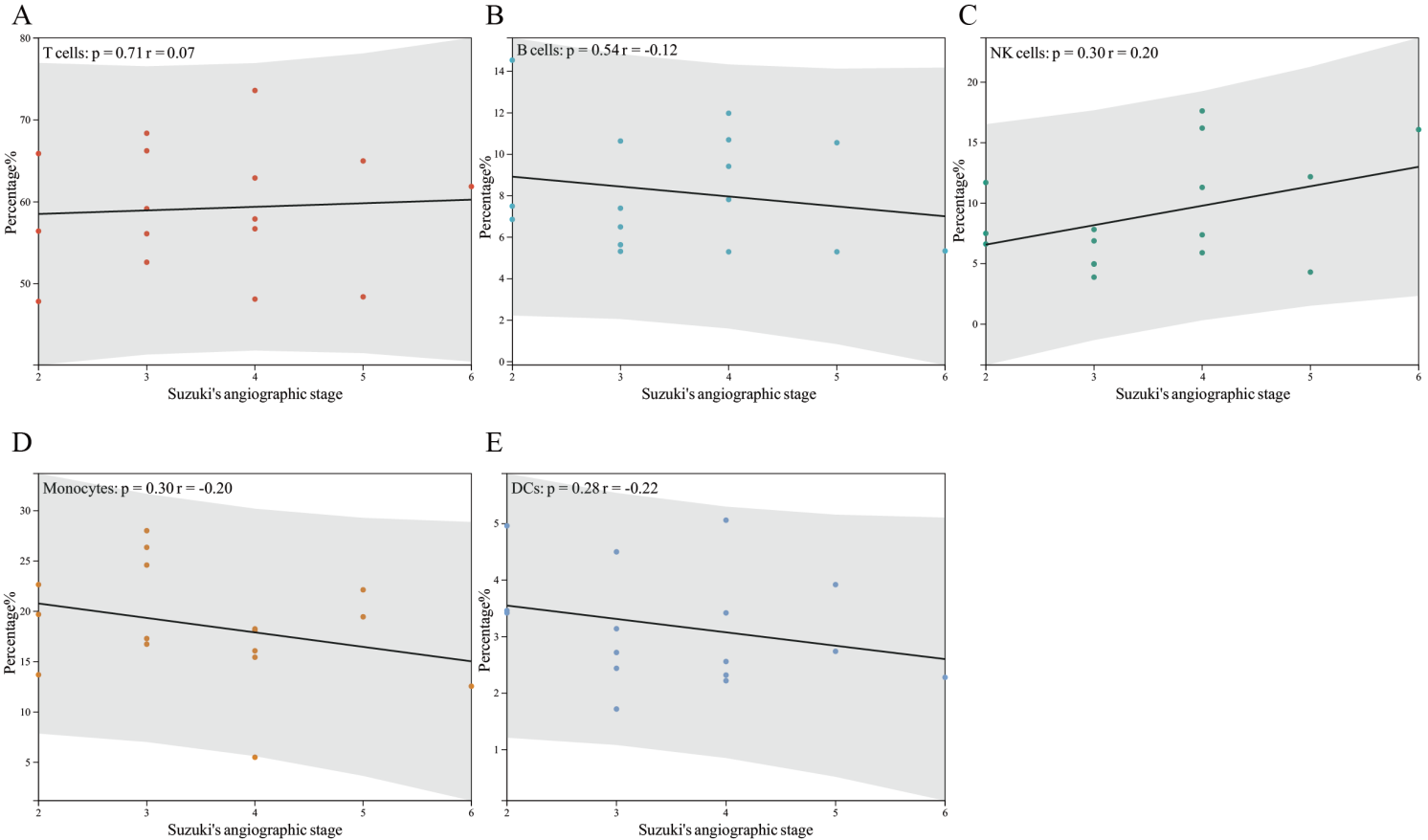
**

**Supplementary Figure 1**. **Kendall Correlation Analysis between Suzuki's Angiographic Stages and the Proportions of Five Cell Clusters.** **(A)** Kendall correlation between Suzuki's angiographic stages and the proportions of T cells. **(B)** Kendall correlation between Suzuki's angiographic stages and the proportions of B cells. **(C)** Kendall correlation between Suzuki's angiographic stages and the proportions of NK cells. **(D)** Kendall correlation between Suzuki's angiographic stages and the proportions of monocytes. **(E)** Kendall correlation between Suzuki's angiographic stages and the proportions of DCs. Statistical significance was set at *P* < 0.05.

**
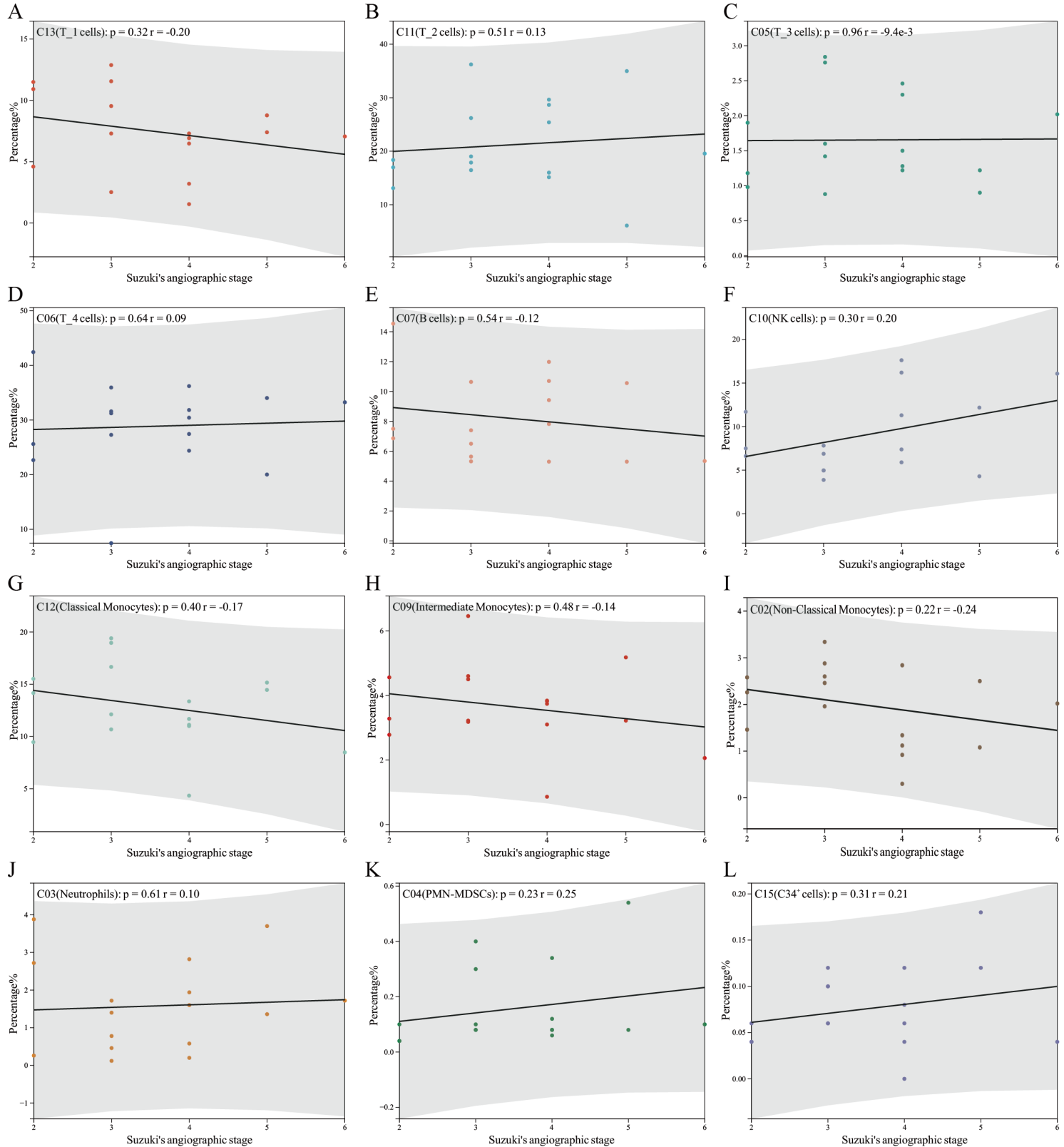
**

**Supplementary Figure 2**. **Kendall Correlation Analysis between Suzuki's Angiographic Stages and the Proportions of Diverse Immune Cell Clusters.** The figure illustrates the Kendall correlation coefficients between Suzuki's angiographic stages and the proportions of different cell clusters (besides DCs Clusters). Notable correlations include **(A)** C13(T_1 cells), **(B)** C11(T_2 cells), **(C)** C05(T_3 cells), **(D)** C06(T_4 cells), **(E)** C07(B cells), **(F)** C10(NK cells), **(G)** C12(Classical Monocytes), **(H)** C09(Intermediate Monocytes), **(I)** C02(Non-Classical Monocytes), **(J)** C03(Neutrophils), **(K)** C04(PMN-MDSCs), and **(L)** C15(C34^+^ cells). Statistical significance was set at *P* < 0.05.
